# Dampened HPG axis activity and altered ovarian gene transcription in Dummerstorf high-fertility mouse line FL2

**DOI:** 10.1530/EC-26-0242

**Published:** 2026-07-15

**Authors:** Carolin L M Ludwig, Simon Bohleber, Anja Baufeld, Eva K Wirth, Martina Langhammer, Ulrich Schweizer, Marten Michaelis, Joachim M Weitzel

**Affiliations:** ^1^ Research Institute for Farm Animal Biology (FBN), Dummerstorf, Germany; ^2^ Institut für Biochemie und Molekularbiologie (IBMB), Uniklinikum, Universität Bonn, Bonn, Germany; ^3^ Charité – Universitätsmedizin Berlin, Corporate Member of Freie Universität Berlin, Humboldt-Universität zu Berlin, Department of Endocrinology and Metabolism, Berlin, Germany; ^4^ DZHK (German Centre for Cardiovascular Research), Partner Site Berlin, Berlin, Germany

**Keywords:** reproduction, high fertility, HPG axis, outbred mouse line, ovary

## Abstract

Animal models of enhanced fertility are rare, as most genetically modified mouse models with reproductive phenotype display subfertility or infertility. Here, we describe the ovarian phenotype of the Dummerstorf line 2 (FL2) mouse strain, which exhibits high fertility and has been selectively bred for increased fertility over more than 190 generations. This long-term selection, outbred mouse line almost doubled the litter size to 21.5 (FL2) compared with 11.3 (unselected control line, ctrl), without showing any signs of growth retardation in the offspring. Here, we show that FL2 females ovulate 25.0 oocytes per cycle compared with 13.2 in ctrl. FL2 mice remain in the estrus phase for a shorter period during a 12-day observation period. Follicle-stimulating hormone (FSH) levels are decreased, both in estrus and diestrus, compared with ctrl, whereas luteinizing hormone levels are unaffected. The mRNA expression levels in the pituitary gland correspond to the gonadotropin levels in the blood. Progesterone levels are decreased in estrus in FL2. Hypothalamic expression levels of gonadotropin-releasing hormone (GnRH) are decreased in diestrus. Holistic gene expression analysis indicates complex and differential regulation in estrus and diestrus in ovaries of FL2 compared with ctrl. In particular, genes of the TGF-β pathway (such as *Bmp3, Bmp7, and Inhba*) and the Wnt pathway (such as *Sfrp4 and Mkrn1*) are differentially expressed in ovaries of FL2 females. These data indicate that reduced activity of the hypothalamic–pituitary–gonadal axis (in particular, lower levels of GnRH, FSH, and progesterone), combined with altered gene transcription in the ovaries, leads to higher ovulation rates in order to achieve the breeding objective of improved fertility.

## Introduction

Research in the field of reproductive biology largely depends on informative animal models. There are approximately 2,500 mouse models annotated with a reproductive phenotype worldwide (https://www.informatics.jax.org/). Interestingly, the vast majority (∼99%) of these mouse lines show a decreased fertility phenotype, characterized by sub- or infertility in one or possibly both sexes. This is counterintuitive to what is intended in human reproductive biology (with the exception of the development of contraceptives). The human birth rate has been declining for decades for a variety of biological and socio-economic reasons (1). In addition, in economically important farm animals (e.g. pigs, bovine, and rabbits), breeding programs largely focus on improving fertility (https://agbu.une.edu.au/breedingfocus.html). Thus, animal models, which show pathways and mechanisms predisposing for improved fertility, might be an important source of information.

However, the number of genetic models with an increased fertility phenotype is very limited. These models are generally transgenic mice in which a single gene or a small number of genes have been modified, resulting in a moderate increase in litter size of around 10–20%. One example is the *Bcl2*-transgenic mouse. In these mice, overexpression of the anti-apoptotic factor *Bcl2* leads to higher litter sizes due to decreased apoptosis in the ovaries (2). Furthermore, the litter size of transgenic mice overexpressing the stem cell factor *Lin28* is markedly larger (3). Although these two models are characterized by larger litter sizes at a young age, fertility declines prematurely in later life. Both lines are not able to maintain high litter sizes (2, 3).

In contrast to classical, inbred mouse lines, attempts have been undertaken to select for increased fertility via selective breeding. The group of Odd Vangen at the Agricultural University of Norway in Ås selected a mouse line for improved fertility for more than 100 generations. This mouse line almost doubled the number of offspring per litter (4). Based on this outbred mouse line, the inbred mouse QSi5 has been developed, which maintained an improved fertility (5). Using a very similar breeding approach, two highly fertile mouse lines (fertility lines 1 and 2; FL1 and FL2) were developed at the FBN Dummerstorf through selective breeding from a heterogeneous founder population. During the selection process, both FL1 and FL2 almost doubled the number of offspring per litter. It should be particularly emphasized here that the Dummerstorf high-fertility mouse lines (FL1 and FL2) show no signs of health problems neither in the mother nor in the offspring (6).

Interestingly, although FL1 and FL2 have been selected independently from the same founder population according to the same selection criteria (high litter size and high total litter weight at first delivery), FL1 and FL2 mice differ in several physiologic and behavioral characteristics. Furthermore, both fertility lines differ from the unselected control mouse line (ctrl). The body weight at 9 weeks of age is higher in both fertility lines compared with the ctrl line, and behavior and fitness tests revealed differences between mouse lines (7). It is interesting to note that life expectancy differs between the FL1 and FL2 lines. Although there is no difference in life expectancy between the control line and FL1, male and female FL2 animals have a significantly shorter life expectancy (−20% shorter) than ctrl (7). Furthermore, the reproductive lifespan differs between the two fertility lines. While the reproductive lifespan of FL1 females (5.4 litter per dam over their lifetime) is similar to that of control mice (6.1 per dam), FL2 females gave birth to a significantly lower number of litter (2.7 per dam) in a long-term breeding experiment (6).

Therefore, both lines developed different molecular adaptations in order to fulfill the breeding goal of improved fertility. In previous work, we described the ovarian phenotype of FL1 females (8, 9). In the present work, we focused on the phenotype of FL2 females. We counted the number of ovulated oocytes per cycle, analyzed the stages of ovarian cycle, examined the parameters of the HPG axis, and performed a holistic gene expression approach in the ovary of cycle-controlled mice in estrus and diestrus to obtain global insights into the development of the high-fertility phenotype in FL2.

## Materials and methods

### The Dummerstorf high-fertility mouse line 2

The Dummerstorf control mouse line (ctrl) and the Dummerstorf high-fertility mouse line 2 (FL2) were originally derived from the same genetic background via selective breeding. Both mouse lines are descendants of the same founder population, which was created by cross-breeding of four different inbred (CBA/Bln, AB/Bln, C57BL/Bln, and XV11/Bln) and four outbred mouse lines (NMRI orig, Han:NMRI, Han:CFW, and Han:CF1) (10, 11). The ctrl line did not undergo any selection process. However, a rotational mating scheme with 125–200 breeding pairs per generation has been used to minimize the degree of kinship. The FL2 line was selected for litter size and the total weight of the litter at birth to primiparous females (Dummerstorf fecundity index = 1.6 × litter size + total weight of the litter in grams) for the first 162 generations. The litter weight was integrated into the Dummerstorf fecundity index to avoid decreased individual birth weight and therefore reduced fitness and growth retardation of the offspring. From the 163rd generation onward, FL2 mice were selected for the best linear unbiased prediction breeding value estimation, focusing only on the litter size. In total, between 60 and 100 breeding pairs of the FL2 line were kept per generation (10). Table 1 shows the body mass, litter size, total litter weight, and weight per pup of FL2 mice and ctrl mice of the indicated generation used in this study.

**Table 1:** Body mass, total litter size at birth, litter weight at birth, and mean birth weight per pup of the mouse generations used in this study.

	Generation number	Body mass at day 63 (g)		Total litter size at birth		Litter weight at birth (g)		Mean birth weight per pup (g)	
		*n*	Mean ± SD	*n*	Mean ± SD	*n*	Mean ± SD	*n*	Mean ± SD
*ctrl*	192	301	31.0 ± 2.9	119	11.3 ± 2.9	119	19.8 ± 4.7	119	1.79 ± 0.2
*FL2*	192	147	41.2 ± 3.1	54	21.5 ± 3.4	54	40.2 ± 5.9	54	1.87 ± 0.2

### Animals and housing

All procedures were performed following national and international guidelines and approved by the institutional board (Animal Protection Board from the Research Institute for Farm Animal Biology). Female mice, bred at the Research Institute for Farm Animal Biology (FBN), Dummerstorf, Germany, were housed in groups of predominantly three animals per cage. A commercial breeding diet (#V1124-300, ssniff, Germany) for rodents and water was provided *ad libitum*. The illumination of animal facilities was between 06:00 and 18:00 h. In addition, a male mouse was kept for acoustic, visual, and olfactory stimulus in a separate cage.

### Determination of the estrous cycle

The estrous cycle of ctrl and FL2 mice was determined as previously described (8, 9). There are various references on the implementation and evaluation of vaginal cytology in mice (12, 13). At the age of approximately 65 days, the estrous cycle of 18 ctrl and 18 FL2 females was determined daily at 09:00 h for a period of 12 days. For the evaluation of the estrous cycle, a PBS drop (Roti®-CELL PBS, Carl Roth GmbH + Co. KG, Germany) was placed at the opening of the vaginal canal and was pipetted up and down three to four times, without penetrating or injuring the vagina. Then, the recovered PBS drop containing flushed cells of the vaginal mucosa was placed on a glass slide and was immediately evaluated under a light microscope (Nikon ECLIPSE TE2000-S, 10x magnification, Japan). The presence, absence, density, and arrangement of the cells indicate the stage of the estrous cycle of the mouse. The murine estrous cycle takes 4 to 5 days and is generally divided into four stages: proestrus, estrus, metestrus, and diestrus. During the estrous cycle, female mice are subject to major changes, which occur at different levels. Cycle-related regulation of gene expression, hormonal alterations in the HPG axis, or anatomical changes in several tissues may affect results of research. Therefore, samples were taken in estrus and diestrus. To keep the time point of sampling within estrus or diestrus as constant as possible, samples were taken when exclusively cornified epithelial cells (no other cells) appeared in vaginal smears in estrus, and only leukocytes appeared in diestrus.

### Sample procedure

The animals, aged approximately 77 days, were euthanized by CO_2_ inhalation between 10:00 and 11:00, after the phase of the estrus cycle had been determined at 9:00. After determination of death, the thorax was opened. The *vena cava caudalis* was cut, and blood was collected out of the thorax cavity. Blood samples were left for two hours at room temperature. The resulting blood clot was removed, and the supernatant was centrifuged (4°C, 2,000 *g*, 10 min) to obtain serum. Both ovaries were extracted, cleaned from fat tissue, and weighed. *Tunica albuginea* and oviduct were removed. In estrus, the cumulus oocyte complex was obtained from the opened oviduct. The oocytes were isolated and counted. Each ovary was weighed. In addition, the uterus was cleaned from fat tissue and weighed. Total pituitary and total hypothalamus were extracted. All samples were immediately snap-frozen in liquid nitrogen and stored at −70°C.

### Reverse transcription qPCR

Hypothalamus samples of 10 animals per line (estrus *n* = 5; diestrus *n* = 5) and pituitary samples of 18 animals per line (estrus *n* = 9; diestrus *n* = 9) were used for qPCR analysis of *Gnrh* (hypothalamus), *Fshb,* and *Lhb* (pituitary). Hypothalamus and pituitary were pulverized in liquid nitrogen. RNA was extracted with an RNeasy Plus Micro Kit (Qiagen, Germany), and 400 ng total RNA was reverse-transcribed using an iScript cDNA Synthesis Kit (Bio-Rad, Germany) according to the manufacturer’s protocol in 20 μL reaction volume. Primers were designed using Primer-BLAST and purchased from TIB Molbiol (Germany). Each sample was prepared using 4 μL of primer mixture (final concentration: 0.2 μM per primer in the PCR mixture), 5 μL of iQ SYBR Green Supermix (Biorad, Germany), and 1 μL of cDNA reaction solution (a 1:10 dilution of the cDNA reaction mixture mentioned above) (total volume of the PCR: 10 μL), onto a 96-well plate and amplified by real-time PCR (iCycler, Biorad, Germany). The quality of the amplification products was assessed using melting curve analysis. In addition, the PCR products of *Gnrh, Fshb, and Lhb* were cleaned using the NucleoSpin® Gel and PCR Clean-up Kit (Macherey-Nagel, Germany) and used as the standard curve (100 fg/μL – 0.1 fg/μL) on which the total mRNA abundance in the samples was determined. The results of the mRNA abundance were calculated relative to a combination of the reference genes *Rps18*, *36b4,* and *B2m*. Relative gene expression was calculated using the relative expression software tool (14, 15). The sequences of the primers are shown in Supplementary Fig. 1 (see section on Supplementary materials given at the end of the article).

### Hormonal analysis

Serum samples of 18 animals per line were used for hormonal analysis (estrus *n* = 9; diestrus *n* = 9). For the measurement of follicle-stimulating hormone (FSH) and luteinizing hormone (LH), undiluted serum samples were assayed (repeated measurement) using an MPTMAG assay kit (Merck Millipore, Germany) on a Luminex LX200 system according to the manufacturer’s instructions. According to the manufacturer, the sensitivity of the assay is 9.5 pg/mL for FSH and 4.9 pg/mL for LH. For both FSH and LH, the intra-assay variation is below 15% and inter-assay variation is below 20% as given by the manufacturer.

Serum concentrations of progesterone (P4) were determined in duplicate using a competitive single-antibody ^3^H-radioimmunoassay (RIA) with [1,2,6,7-^3^H] progesterone tracer (Hartmann Analytic, Germany) in duplicate, as previously described (16, 17). Radioactivity was counted using a liquid scintillation counter with an integrated RIA program (TriCarb 2900 TR; Perkin-Elmer, Germany). This assay has a sensitivity of 7 pg/mL. The intra-assay variation was 7.6%, and the inter-assay variation was 9.8%.

### Statistical analysis

Statistical analysis of the results of the hormonal analysis and the qPCR were performed with the software GraphPad Prism® (version 5.01). All data points were included in the analysis, and no outlier data points have been omitted. The statistical methods used for the various analyses are specified in the respective figure legends.

### Transcriptome analysis

Samples of 10 animals per line (estrus *n* = 5; diestrus *n* = 5) were used for the transcriptome analysis. RNA was extracted using the RNeasy Plus Micro Kit (Qiagen, Germany). Libraries were prepared with the QuantSeq 3′ mRNA-Seq Fw. Library Prep kit (Lexogen, Austria) and sequenced to 10M raw reads on an Illumina HiSeq 2500 machine, following the manufacturer’s protocols. Data were pre-processed and analyzed using the options recommended by the manufacturer. Trimmed sequences were aligned with STAR aligner 2.6.0a (18) against the GRCm38 (release 95) mouse genome retrieved from the Ensembl database via biomaRt (19) tool. Raw sequence data and raw counts were deposited at the NCBI GEO repository, entry GSE32554. Differential expression analysis was performed with the DESeq2-package v1.14.1 (20) in R 4.4.2. Hierarchical clustering, in combination with a corresponding heatmap illustrating the differentially expressed genes (DEGs), was performed using the mixOmics package in R (21).

### Re-evaluation of the transcriptome analysis with qPCR

Six (estrus) and five (diestrus) DEGs were selected on the basis of their involvement in a described fertility phenotype and analyzed using qPCR in order to re-evaluate the results of the transcriptome analysis (biological replicates). The selection criteria were chosen at random from the following groups: genes that were expressed differently during both estrus and diestrus (*Bmp7, Rpl29, and Efemp1*); genes that were described in more detail in connection with the FL2 phenotype (*Inhba, Cxcr4, and Igfbp2*); and genes that were expressed in both high-fertility lines, FL1 and FL2 (*Tex14, Igfbp2, Rpl29, and Cxcr4*). For this validation experiment, 20 animals per line (estrus *n* = 10; diestrus *n* = 10) were used. The RNA was extracted, reverse-transcribed, amplified by PCR, and analyzed as described above. Each sample was analyzed in duplicate (technical replicates). The sequences of the primers are shown in Supplementary Fig. 1.

## Results

### Ovulation rate and phenotypic description

**Figure 1:**
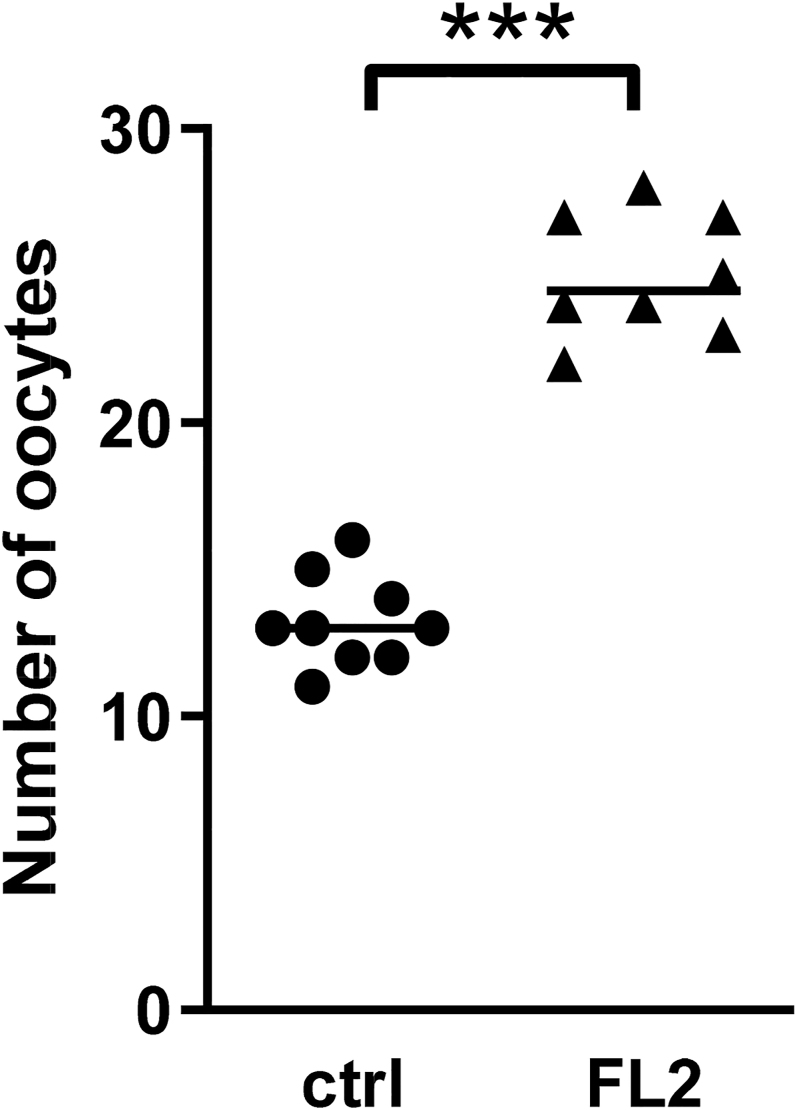
Number of ovulated oocytes in ctrl mice and FL2 mice. The oocytes were isolated out of the cumulus in the oviduct in cycle-controlled animals in estrus. Data are expressed as mean ± S.E.M., ***P < 0.001 (two-tailed *t*-test).

During the selection process, the number of offspring per litter in the FL2 females almost doubled, from 11.3 ± 2.9 (ctrl) to 21.5 ± 3.4 (FL2), without the offspring showing any signs of growth retardation (Table 1). Ctrl females ovulated 13.2 ± 0.5 (mean ± SEM) oocytes, whereas FL2 females ovulated 25.0 ± 0.8 oocytes (Fig. 1).

To further characterize FL2 female mice, the weights of the reproductive organs were analyzed. We found slightly decreased ovarian and uterus weights in diestrus but not in estrus in FL2 compared with ctrl (Table 2). However, it should be noted that the uterus weight differs greatly by approximately 50% between estrus and diestrus in both lines (Table 2).

**Table 2:** Weight (mean, ±SD) of reproductive organs of ctrl and FL2 mice in estrus and diestrus.

	Ovary (g)		Uterus (g)	
	Estrus	Diestrus	Estrus	Diestrus
*Ctrl*	0.039 ± 0.017	0.036 ± 0.006	0.187 ± 0.040	0.115 ± 0.016
*FL2*	0.035 ± 0.007	0.028^†^ ± 0.003	0.157 ± 0.016	0.101* ± 0.009

^*^ P < 0.05

^†^ P < 0.01

**Figure 2:**
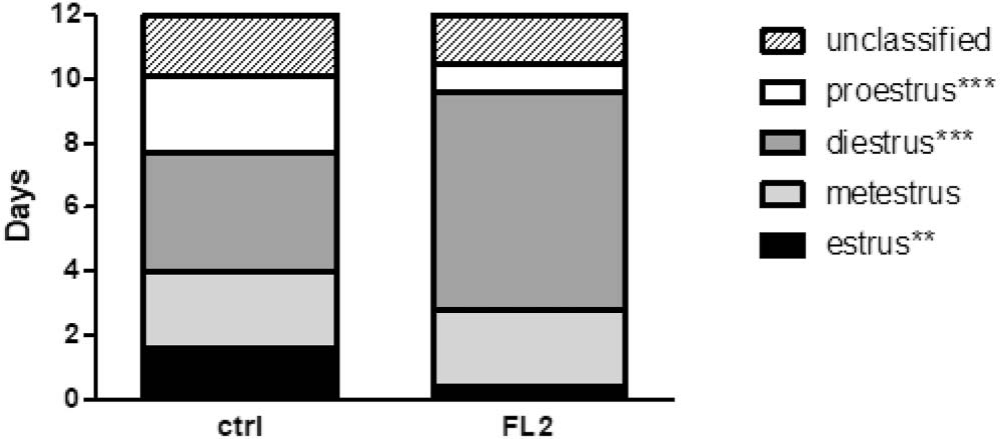
Distribution of the total time of the stages of estrous cycle in ctrl mice and FL2 mice over a time period of 12 days. Data are expressed as means of the timeframes in the different stage of estrous cycle. The distribution of the stages of estrous cycle was significantly different between ctrl mice (*n* = 18) and FL2 mice (*n* = 18) in estrus, diestrus, and proestrus, **P < 0.01, ***P < 0.001 (Welch’s test).

The estrous cycle was monitored over a 12-day observation period (Fig. 2). The distribution of the stages of estrous cycle was significantly different between ctrl and FL2 mice. During the observation period of 12 days (=100%), estrus was determined in ctrl mice in 1.7 ± 0.3 days (14%) compared with 0.4 ± 0.2 days in FL2 mice (3%). Thus, the duration of the estrus phase during the observation period was significantly shorter (*P* = 0.004) in FL2 mice compared with ctrl mice. Metestrus was determined in 2.4 ± 0.3 days (20%) in ctrl mice and 2.4 ± 0.3 days (20%) in FL2 mice. We observed ctrl mice remain 3.7 ± 0.5 days and FL2 mice 6.8 ± 0.4 days in diestrus, which corresponds to 31% (ctrl) and 56% (FL2). Thus, FL2 mice remain significantly (*P* < 0.001) longer in diestrus than ctrl mice. Finally, times in proestrus differ from 2.4 ± 0.4 days (20%) in ctrl mice to 0.9 ± 0.2 days (8%) in FL2. Thus, the proestrus phase is significantly (*P* < 0.001) shorter in FL2 mice compared with ctrl mice.

### Expression of the hypothalamic hormone GnRH

**Figure 3:**
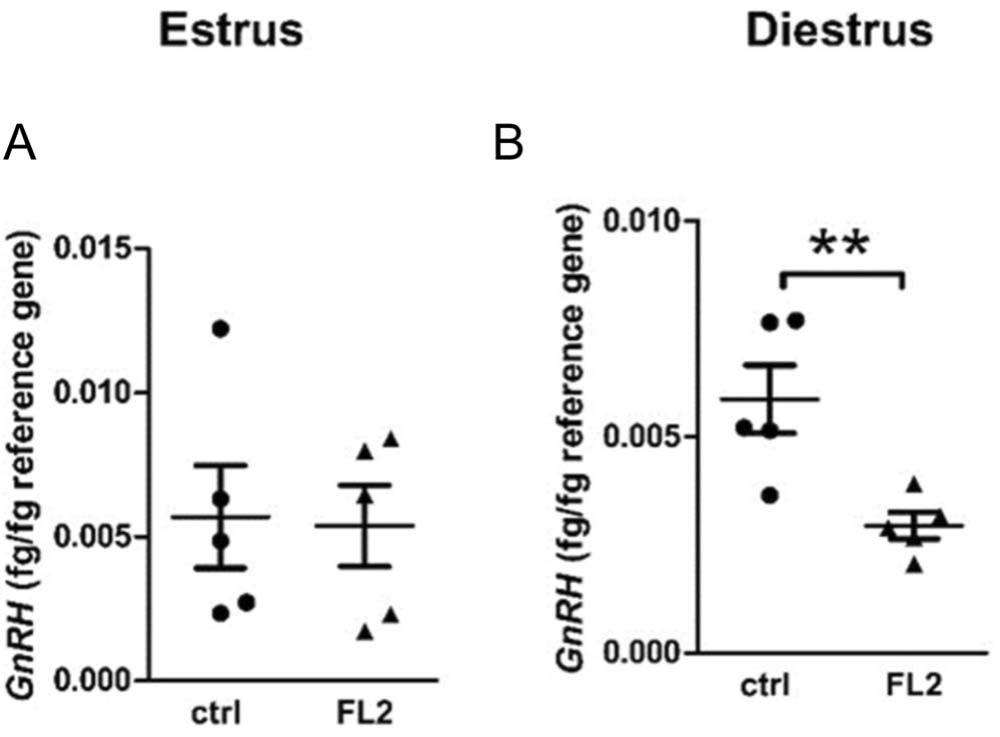
mRNA content of the hypothalamic hormone *GnRH* in ctrl mice and FL2 mice in estrus and diestrus. *GnRH* mRNA content (fg/fg reference gene) in estrus (A) and diestrus (B). Data are expressed as mean ± S.E.M, **P < 0.01 (Mann–Whitney U test).

*GnRH* was analyzed at the transcript level from hypothalamic RNA (Fig. 3A and B). We observed significantly lower transcript levels in FL2 during diestrus compared with ctrl, while transcript levels in estrus remained unchanged in ctrl compared with FL2 (Fig. 3).

### Expression and serum concentrations of pituitary hormones

**Figure 4:**
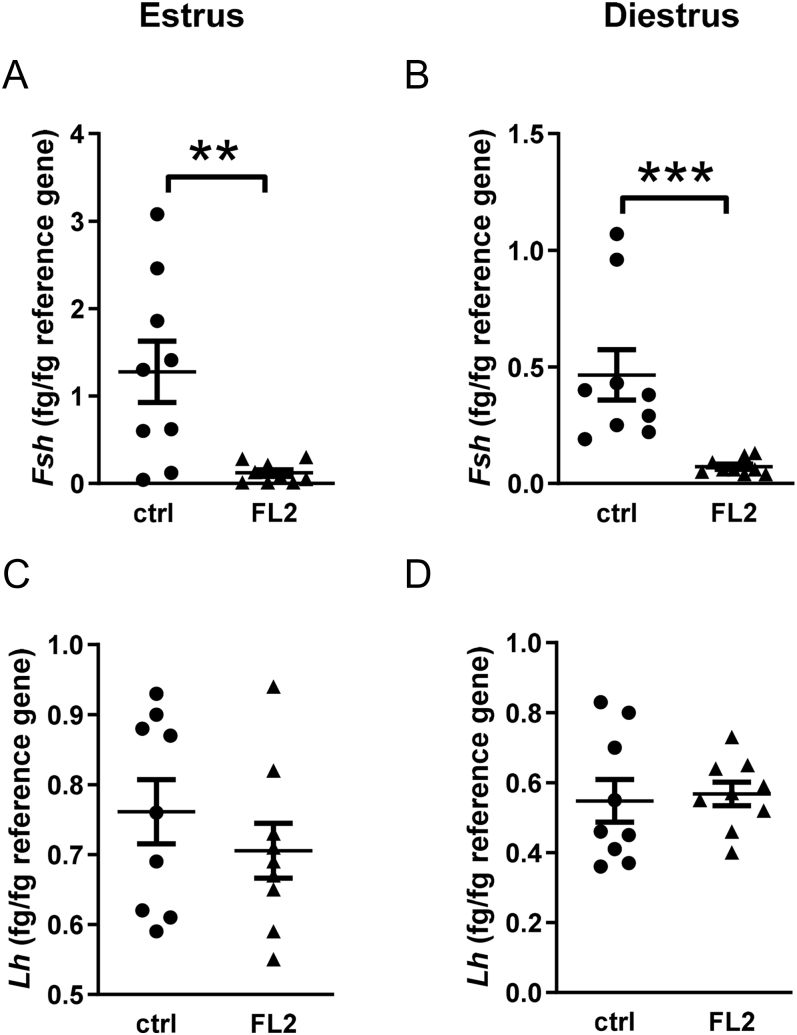
mRNA content of the pituitary hormones *Fshb* and *Lhb* in ctrl mice and FL2 mice in estrus and diestrus. *Fsh* mRNA content (fg/fg reference gene) in estrus (A) and diestrus (B). *Lh* mRNA content (fg/fg reference gene) in estrus (C) and diestrus (D). Data are expressed as mean ± S.E.M, **P < 0.01, ***P < 0.001 (Mann–Whitney U test).

**Figure 5:**
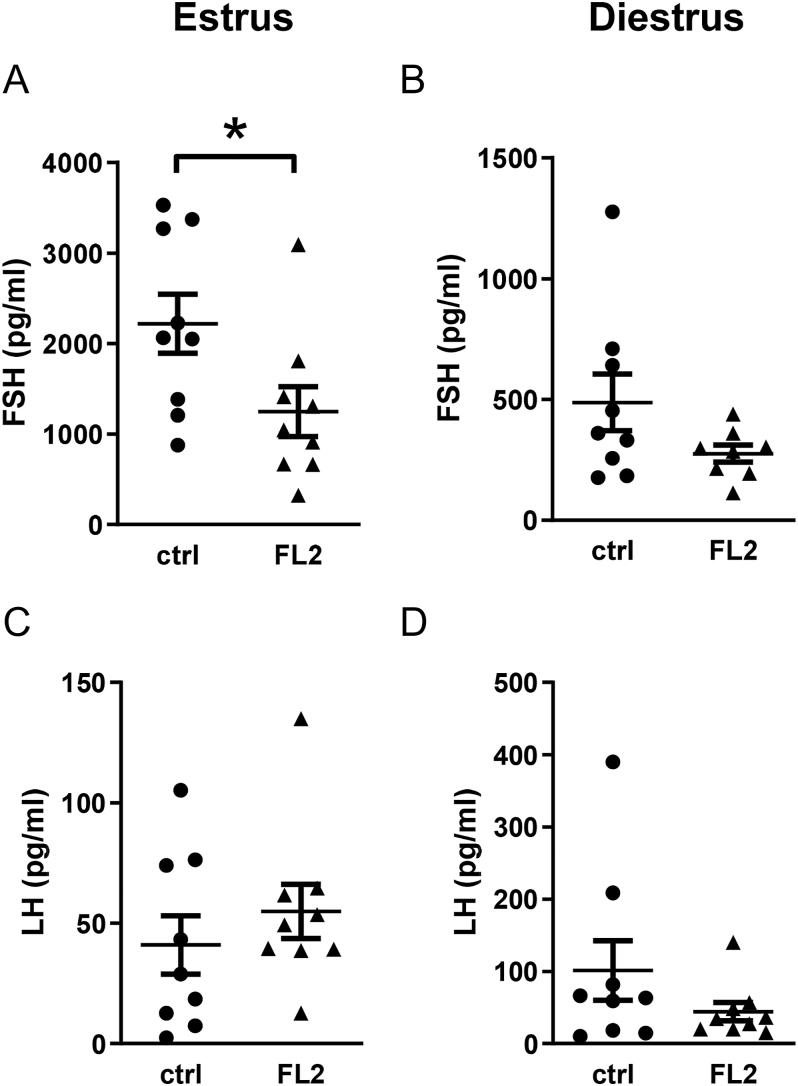
Serum concentration of the pituitary hormones FSH and LH. Serum concentration of FSH (pg/mL) in ctrl mice and FL2 mice in estrus (A) and diestrus (B). Serum concentration of LH (pg/mL) in ctrl mice and FL2 mice in estrus (C) and diestrus (D). Data are expressed as mean ± S.E.M, *P < 0.05 (Mann–Whitney U test).

FSH was analyzed at both transcriptional (Fig. 4A and B) and protein (hormone) levels (Fig. 5A and B). Overall, we found a significant reduction in FSH protein and transcript levels in FL2 mice compared with ctrl mice. FSH hormone concentrations were significantly lower in FL2 mice (1,248.0 ± 274.9 pg/mL) compared with ctrl mice (2,220.2 ± 327.4 pg/mL) in estrus (*P* = 0.04, unpaired *t*-test). In diestrus, the FSH concentrations in serum were 275.7 ± 35.9 pg/mL in FL2 mice and 488.1 ± 116.6 pg/mL in ctrl mice. At the mRNA level in the pituitary, we detected significantly lower *Fshb* mRNA content in both estrus and diestrus of FL2 compared with ctrl (Fig. 4A and B). In addition, LH levels were analyzed at both transcriptional (Fig. 4C and D) and protein (Fig. 5C and D) levels. We found no significant difference in LH levels between FL2 and ctrl mice.

### Concentration of progesterone

**Figure 6:**
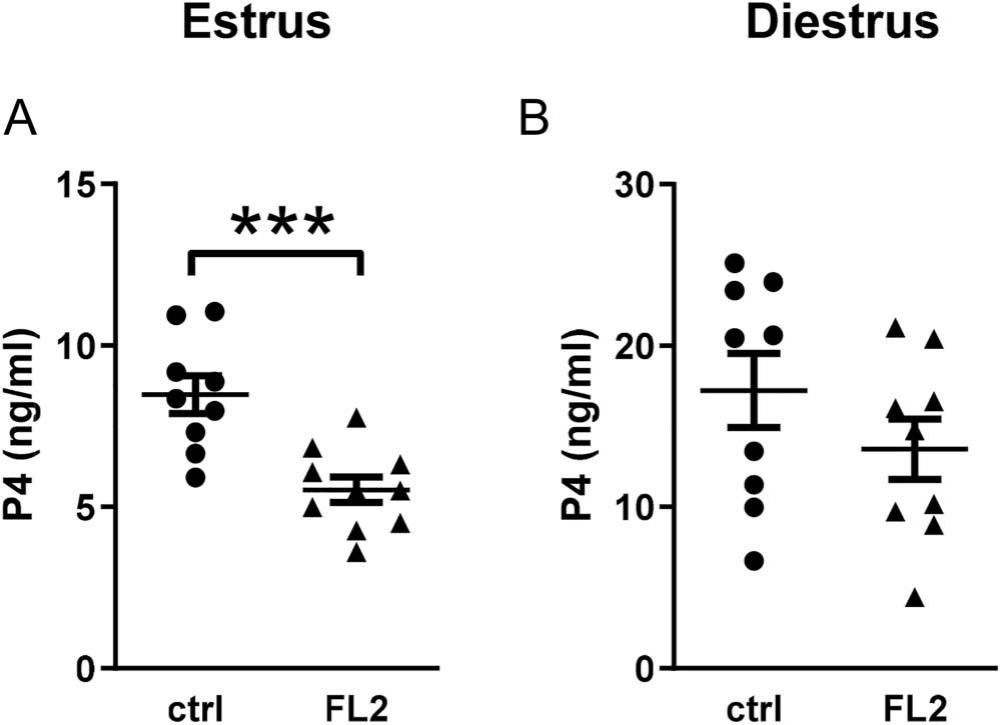
Serum concentration of P4 (ng/mL) in ctrl mice and FL2 mice in estrus (A) and diestrus (B). Data are expressed as mean ± S.E.M, ***P < 0.001 (Mann–Whitney U test).

Progesterone (P4) levels were analyzed in the serum (Fig. 6A and B). In estrus of FL2, we found significantly lower P4 serum concentrations (5.5 ± 0.4 ng/mL) compared with ctrl (8.5 ± 0.6 ng/mL). We found no significant difference in P4 concentration between FL2 (13.6 ± 1.9 ng/mL) and ctrl (17.2 ± 2.3 ng/mL) in diestrus.

### Transcriptome analysis and re-evaluation with qPCR

**Figure 7:**
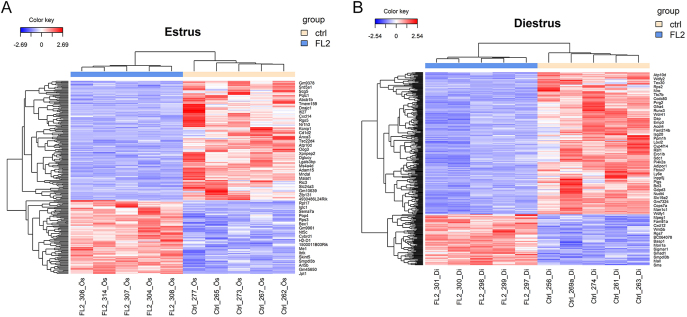
Hierarchical clustering dendrograms of DEGs (log2FC) in (A) estrus and in (B) diestrus of FL2 and ctrl mice. High and low expression intensities are represented by red and blue color, respectively (see scale on the left margin).

A transcriptome analysis of estrus and diestrus from ctrl and FL2 mice revealed line-specific expression patterns. Compared with ctrl mice, 233 genes were differentially expressed (89 higher and 144 lower expressed genes with *q* ≤ 0.05) in estrus, while 341 genes were differentially regulated (90 higher and 251 lower expressed genes with *q* ≤ 0.05) in diestrus of FL2 mice (Fig. 7). Genes that are associated with reproductive traits are shown in Table 3 (estrus) and Table 4 (diestrus). Both lists of DEGs share five common genes (*Rpl29, Efemp1, Bmp7, Cobll1, and Nr1h3*), which are differently expressed both in estrus and in diestrus, whereas other genes are regulated in either the estrus or diestrus of FL2 females (Tables 3 and 4).

**Table 3:** List of genes associated with reproductive traits in estrus. The ΔLog2FC is calculated based on the result of the transcriptome analysis (*q < 0.05, **q < 0.01, ***q < 0.001, *n* = 5). In addition, the ΔLog2FC based on the results of the qPCR validation experiment is shown (*n* = 10).

Gene	Function	Log2FC	Log2FC qPCR	Effects on reproduction
Nr5a2	Nuclear receptor	0.67*		Reduced female fertility, increased uterus weight, decreased progesterone levels, and abnormal pregnancy (30)
Cobll1	Cofactor AR	−1.12***		Abnormal ovarian morphology (37)
Nr1h3	Nuclear receptor	−1.2*		Ovary cyst, abnormal corpus luteum morphology, abnormal ovary physiology, enlarged ovary, abnormal superovulation (31), and decreased LH levels (32)
Bmp7	TGF-β signaling	−2.41***	−1.5***	Uterine receptivity and blastocyst attachment (38)
Porcn	Modulator of Wnt signaling	2.00***		Altered uterine development (39)
Aldh2	Protect against ox stress	−0.38*		Litter size (40)
Por	Cytochrome P450 oxidoreductase	−0.57*		Estrus cycle disorders and female infertility (41)
Bmp3	TGF-β signaling	1.19**		CL development (42)
Caecam10	Cell adhesion molecule	0.39***		Litter size (43)
Slc7a5	AS transporter regulates mTOR pathway	−2.28***		Gonadal fat pad weight (44)
Rpl29	Ribosomal protein	−5.5***	−5.6***	Delayed sexual maturation (45)
Igfbp2	IGF binding	−0.91**	−0.45*	Follicular development (46) and follicular atresia (47)
Efemp1	Extracellular matrix	−1.84***	−1.44***	Fertility, litter size, and premature aging (48)
Tex14	Intercellular bridges	1.52**	0.67*	Reproductive lifespan (49)
Inhba	Activin/inhibin and TGF-β superfamily	1.79*	1.92***	Ovary size, FSH levels, number of mature ovarian follicles, and altered estradiol and progesterone levels (28)
				Fertility, litter size, number of corpora lutea, and polyovular follicles (29)
Cxcl14	Chemokine and apoptosis	−0.99*		Decreased progesterone production and decreased luteinized granulosa cells (50)

**Table 4:** List of genes associated with reproductive traits in diestrus. The ΔLog2FC is calculated based on the result of the transcriptome analysis (*q < 0.05, **q < 0.01, ***q < 0.001, *n* = 5). In addition, the ΔLog2FC based on the results of the qPCR validation experiment is shown (*n* = 10).

Gene	Function	Log2FC	Log2FC qPCR	Effects on reproduction
Rpl29	Ribosomal protein	−6.15***	−5.94***	Delayed sexual maturation (45)
Rps2	Component of the ribosome	−5.45***		Steroid hormone sensing (51)
Efemp1	Extracellular matrix protein	−2.61***	−1.66***	Fertility, litter size, and premature aging (48)
Gabrb1	Gamma-aminobutyric acid (GABA) A receptor	−2.53***		Fertility, corpus luteum morphology, altered FSH, LH, GH, and PRL levels (52)
Bmp7	TGF-β signaling	−2.44**	−1.47**	Uterine receptivity and blastocyst attachment (38)
Akr1c18	Conversion of progesterone	−2.40**		Litter size, progesterone levels, prolongation of estrus cycle, and prolongation of diestrus (33)
Sdhd	Links citric acid cycle and oxidative phosphorylation	−2.00		Oxidative stress, infertility, and miscarriage (53)
F2r	Protease-activated G protein-coupled receptor	−1.77***		Ovarian cancer (54)
Cpeb3	Interacts with actin cytoskeleton	−1.64*		Size and weight of ovary, atrophy of ovary, follicular development, FSH levels, fertility, litter size, number of corpora lutea, estradiol levels, oocyte degeneration, number of all kinds of ovarian follicles, number of atretic ovarian follicles, and granulosa cell proliferation and apoptosis (55)
				Fertility (56)
Cobll1	Cofactor AR	−1.54*		Abnormal ovarian morphology (37)
Mme	Proteinase and extracellular matrix	−1.25		Litter size (57)
Nr1h3	Nuclear receptor	−1.24*		Decreased LH levels (32)
Sfrp4	Blocks Wnt signaling	−1.19***	0.49	Regulation of follicular development, litter size, number of ovarian follicles (58), fertility, and litter size (59)
Bcl3	Stabilizing c-Myc protein via ERK activation and apoptosis	−1.15*		Mammary gland development, reproductive lifespan (60), and ovarian cancer (61)
Raf1	Raf kinase family, proliferation, differentiation, apoptosis, and motility	−0.94*		FSH signaling and steroid hormone production (62)
Npc1	Lysosomal cholesterol exporter	−0.91***		Granulosa cell morphology, number of ovarian follicles, ovarian morphology, presence of corpora lutes, ovary weight, and fertility (63)
Bmp4	TGF-β signaling	−0.78*		CL development (42)
Pi4k2a	Vesicular trafficking	0.66*		Fertility (64)
Smad4	Transcription cofactor	−0.60*		Litter size, folliculogenesis, cumulus expansion, morphology of granulosa cells, morphology of ovarian follicles, number of secondary and tertiary follicles, and number of oocytes (34)
Mkrn1	E3 ubiquitin ligase and regulator Wnt pathway	−0.49*		Circulating FSH and LH levels, estrous cycle, sexual maturation, and vaginal opening, (65)
Igfbp7	IGF binding	−0.49*		Litter size, mammary gland, embryo implantation, and uterine receptivity (66)
Cxcl12	Chemokine	0.80***		Endometrial receptivity and implantation (67)
Smad1	Transcription cofactor	0.99**		Number of primordial germ cells (35, 36, 68, 69), fertility, and litter size (70)
Hmga2	DNA-binding protein and TGF-β signaling	1.18*		Ovarian dysfunction, granulosa cell proliferation (71), reduced reproductive tissue size, and behavior abnormalities (72)
Cxcr4	Cell proliferation and cell migration	1.44*	1.01**	Primordial germ cell migration (73) and granulosa cell survival (73, 74)
Mmp12	Matrix metallopeptidase and tissue remodeling	1.96**		Litter size, implantation, and early pregnancy (75)

The differential expression profiles from both estrus (Table 3) and diestrus (Table 4) reveal a coherent re-wiring of ovarian pathways that underlie the FL2 high-fertility phenotype. Core components of the TGF-β/BMP axis are consistently altered: *Bmp7* is strongly downregulated in both stages (log_2_FC ≈ −2.4), while *Bmp3* is upregulated in estrus and *Bmp4* is modestly reduced in diestrus, and the downstream target *Inhba* is upregulated (log_2_FC ≈ +1.8). Nuclear receptor signaling is also reshaped, with *Nr5a2* induced (log_2_FC +0.67) and *Nr1h3* repressed (log_2_FC ≈ −1.2), a pattern linked to the observed decrease in estrus-phase progesterone. Genes governing protein synthesis are dramatically suppressed, most notably *Rpl29* (log_2_FC −5.5 to −6.2) and *Rps2* (log_2_FC −5.45), reflecting a down-scaled translational apparatus. Several regulators of follicular development and steroid metabolism are differentially expressed: *Cobll1, Efemp1, Igfbp2, Akr1c18, Gabrb1, Cpeb3, Smad4, Mkrn1, and Sfrp4* (the latter a Wnt antagonist) are all downregulated, whereas *Porcn* (Wnt activator) and *Smad1* are upregulated, indicating a shift toward enhanced Wnt signaling. Additional DEGs with reproductive relevance include *Tex14* (↑), *Cxcr4* (↑), *Mmp12* (↑), *Hmga2* (↑), and *Cxcl12* (↑), which together influence germ cell migration, extracellular matrix remodeling, and ovarian cell proliferation. The overlap of five genes (*Rpl29, Efemp1, Bmp7, Cobll1, and Nr1h3*) across both tables underscores a core set of regulators that drive the high-ovulation, yet hormonally dampened, phenotype of FL2.

**Figure 8:**
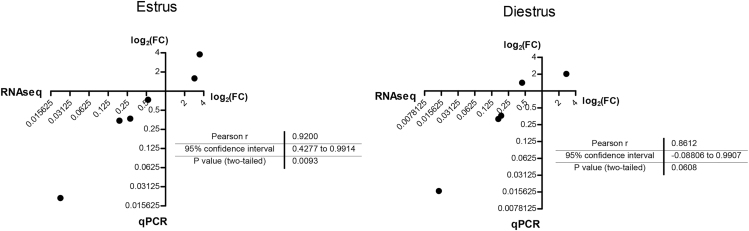
Concordance and correlation of the results of the transcriptome analysis and the qPCR. The log2FC is for mRNAseq, and qPCR for selected genes is shown for samples in estrus (A) and in diestrus (B).

Quantitative PCR on randomly selected genes was used to re-evaluate the transcriptome data in biological replicates (log2FC qPCR, Tables 3 and 4). The correlation between log2FC of the transcriptome sequencing and the qPCR analysis was *r* = 0.92 in estrus and *r* = 0.86 in diestrus cycle stages (Fig. 8).

## Discussion

In this study, we present the activity of the HPG axis and the differences in gene transcription and other physiological characteristics in the ovaries of the Dummerstorf FL2 mouse line, which has been selectively bred for high fertility over a period of 50 years and 192 generations. FL2 mice almost doubled the number of offspring per litter according to the selection criteria from 11.3 (unselected control line) to 21.5 (FL2), respectively (Table 1). Consequently, FL2 females increased the ovulation rate to 25.0 oocytes per ovulation (Fig. 1). A detailed analysis of the different ovarian cycle phases revealed that FL2 females remain in proestrus and estrus for a shorter duration and in diestrus for a longer duration within a 12-day observation window compared with ctrl (Fig. 2) and thus might have less chances to get pregnant. This observation might be connected to a slightly reduced productive mating rate in FL2 (90.1%) compared with ctrl (95.6%); however, it should be considered that these mating rates are still in the upper range compared with productive mating rates in commonly used inbred mouse lines, such as C57BL6 (84%) (6, 22). This raises the question of reasons behind these large differences in the characteristics of the ovarian cycle in FL2 mice. One reason for this could be a mutation in the progesterone receptor (PR) connected to an altered progesterone signaling, which is supported by Palma-Vera *et al.* (23). A whole-genome sequencing analysis on FL2 mice revealed line-specific patterns of genetic variation among the line and found that the PR (*Pgr*) is carrying a missense mutation (23) (see also the discussion below).

Endocrine analysis revealed reduced concentrations of GnRH and FSH at the transcript level (Figs 3 and 4) and in blood hormone levels for FSH (Fig. 5A and B). This observation is counterintuitive to what one would expect, since administration of FSH or substances that simulate FSH effects is widely used to increase ovulation rates in both human and several animals (24, 25, 26). However, systemically elevated levels of FSH seem to be a double-edged sword. As shown by McTavish *et al.*, the permanent and systematic overexpression of FSH in mice leads to an initial higher litter size for the cost of accelerated ovarian failure and a reduced lifetime fecundity (27). Thus, in order to maintain a high fertility, it might be helpful to maintain a reduced set point of FSH concentrations. Alternatively, FSH could be glycosylated in different ways, thereby modulating its bioactivity. Interestingly, the phenotype characterized by reduced FSH levels has also been preserved in FL1 females, a second Dummerstorf mouse strain with high fertility that has been selectively bred for improved fertility (8). Moreover, decreased FSH is even more pronounced in FL1 (FSH concentration in estrus: 788 pg/mL) than in FL2 females (FSH, estrus: 1,248 pg/mL) compared with ctrl females (FSH, estrus: 2,220 pg/mL) (Fig. 5A) (8). It should be considered that FSH concentrations in estrus are reduced by −65% and −45%, respectively, in both high-fertility lines. Similar effects can be observed in diestrus (Fig. 5B). However, the underlying mechanisms that lead to the reduced FSH concentrations in both Dummerstorf fertility mouse lines appear to be regulated differently in FL1 and FL2. While RNAseq data of the ovaries of FL2 mice clearly indicate that multiple genes play an important role in the TGF-β and BMP signaling pathway, such as *Inhba*, and several BMPs and Smads (Tables 3 and 4), these changes could not be observed in the FL1 mice (8). Particular mention should be made here of the role of *Inhba*, a subunit of both activin A and inhibin A, which is expressed at significantly higher levels in the ovaries of FL2 mice (Table 3). The role of *Inhba* is well established as one of the key regulators of FSH levels and is associated with the number and maturation of ovarian follicles and altered progesterone concentrations (28, 29). All these aspects were also noticed in FL2 mice. Preliminary, as yet unpublished data showed that, in 14-day-old animals, the follicular pool was approximately 20% higher in the FL2 group compared with the ctrl group (corresponding to follicles after the cystic breakdown but before the onset of puberty). Consequently, elevated levels of *Inhba*, released from an enlarged follicular pool, may contribute to a downregulation of FSH in these animals.

We did not observe a significant alteration in LH hormone levels in both estrus and diestrus, a feature also observed in FL1 (Fig. 4C and D, Fig. 5C and D) and (8). Furthermore, we observed slightly reduced P4 levels in estrus, in both FL1 and FL2 females (Fig. 6A and (8)). Fittingly, the results of the transcriptome analysis of ovaries of FL2 mice also show that many genes related to steroidogenesis and extracellular matrix are differentially regulated in the ovaries of FL2 mice. One example is *Nr5a2* (formally known as LRH-1), which is higher expressed in the ovaries of FL2 mice in estrus (Table 3). Heterozygous *Nr5a2+/−* mice show a reduced fertility, which is accompanied by reduced serum progesterone levels (30). A second example is the nuclear receptor *Nr1h3*, formally known as *LXRα,* which is lower expressed in the ovaries of FL2 mice in both estrus and diestrus (Tables 3 and 4). *Nr1h3* knockout mice are infertile showing various disturbances of ovarian physiology, including altered ovarian steroidogenesis, abnormal corpus luteum morphology, and abnormal superovulation (31, 32). In addition, the gene *Akr1c18*, which is lower expressed in FL2 mice in diestrus, has been shown to influence progesterone levels. The enzyme converts progesterone to the biologically less active 20-alpha-hydroxy-progesterone (33). Furthermore, *Akr1c18* knockout mice are subfertile, showing a prolonged estrous cycle mainly due to a prolonged diestrus (33), the latter feature also observed in FL2 mice (Fig. 2). Interestingly, a missense mutation within the PR has previously been described by genomic sequencing of FL2 (23). Although the detected point mutation PR-Gly252Glu within the N-terminal AF-1 domain is not predicted to affect progesterone signaling by in silico analysis and we did not observe a differential expression of the PR in the present gene expression analysis (data not shown), the RNAseq data point toward a differential regulation of the progesterone signaling pathway (Tables 3 and 4).

Furthermore, upstream regulators of transcription factors, such as the transcription cofactors *Smad1* and *Smad4,* are differentially expressed in FL2 ovaries compared with ctrl, and interestingly, *Smad1* is one of only five genes (*Smad1*, *Tex14*, *Igfbp2*, *Rpl29*, and *Cxcr4*) that are also differentially regulated in FL1 ovaries (Tables 3 and 4 and (8)). Both Smad cofactors have been shown to have a wide variety of functions in ovaries and folliculogenesis, including alterations of the number of different follicular stages (from primordial germ cells up to secondary and tertiary follicles) (34, 35, 36). Consequently, ovarian-specific *Smad4* knockout mice have been shown to have a reduced number of oocytes accomplished by a reduced litter size (34). It is noteworthy that several members of the Smad family are targets of the TGF-β, Wnt, and BMP signaling pathways, many of which are differentially regulated in FL2 during estrus and diestrus (Tables 3 and 4).

In addition to litter size and the birth of healthy offspring, lifetime reproductive performance is another key aspect of fertility that is important for both humans and economically significant livestock (e.g. pigs). Although the FL2 mice have almost doubled the number of their offspring compared with the unselected control (at first delivery), they cannot maintain this enormous litter performance for long. In a long-term breeding experiment performed by Langhammer *et al.*, FL2 mice had 2.7 litter per dam in a lifetime fecundity experiment. For comparison, ctrl females delivered 6.1 litter per dam and FL1 females 5.4 litter per dam (6). Thus, FL2 females are characterized by a significantly decreased lifetime fecundity compared with ctrl and FL1 females. Considering the short estrus duration of FL2 (Fig. 2), the experimental design of this lifetime fecundity experiment might be unfavorable for the FL2-like fertility phenotype.

**Figure 9:**
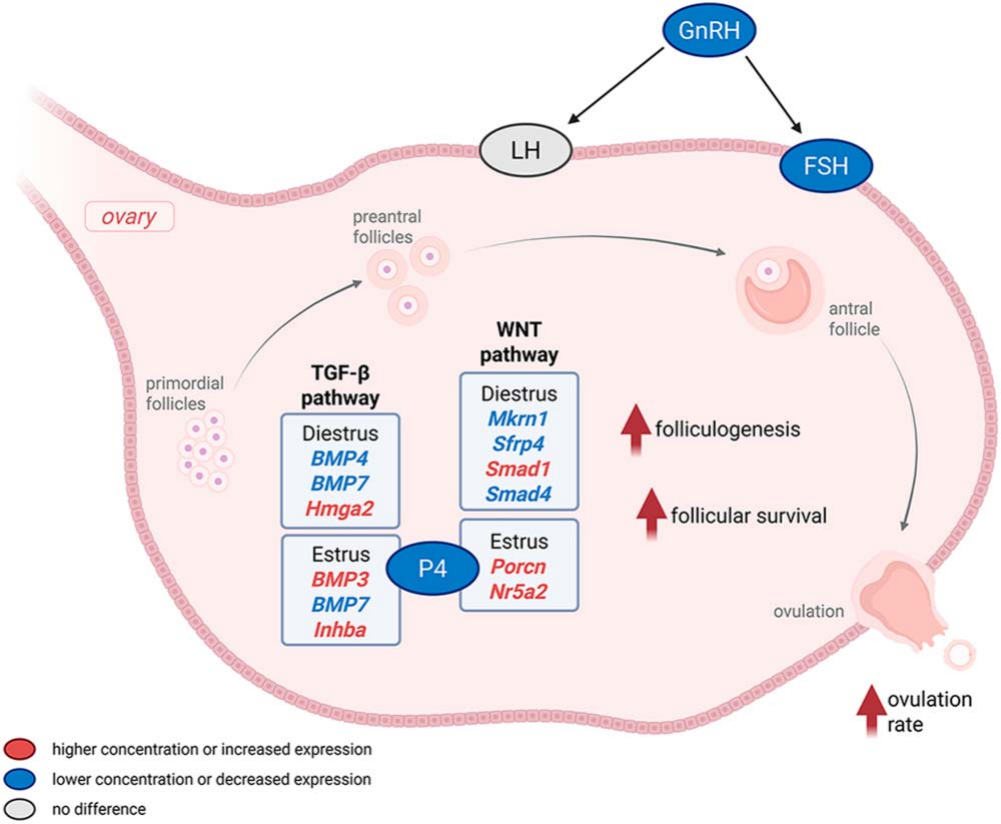
Follicular maturation in FL2 mice. Gene expression data and the measurement of parameters of the HPG axis on endocrine levels indicate that different traits of follicular development are improved in FL2 mice. Our results demonstrate that the increased ovulation rate of FL2 mice is due to a complex interplay of different cycle-related regulation of TGF-β and Wnt pathways, leading to increased folliculogenesis and increased follicular survival (figure created with BioRender.com).

Taken together, our data indicate that FL1 and FL2 developed overlapping and divergent strategies in order to warrant the selection goal of improved fertility via breeding starting from a heterogeneous (and originally identical) gene pool. Common aspects are a dampened HPG axis activity most considerably visible by reduced GnRH and FSH levels and reduced progesterone levels (Figs 3, 4, 5, 6). However, the molecular translation in order to warrant this endocrine phenotype seems to be differently regulated in FL1 and FL2. While FL1 mainly modified the folliculogenesis in the direction of increased follicular survival accompanied by a reduced follicular atresia (8, 9), FL2 mainly modified other endocrine pathways, such as the TGF-β, Wnt, and BMP signal transduction pathways (Tables 3 and 4; graphically summarized in Fig. 9).

Furthermore, these data suggest that a combination of reduced FSH (in both estrus and diestrus), unchanged LH, and reduced P4 (in estrus) appears to be part of the endocrine framework that maintains and ensures a high ovulation rate in mice. However, these endocrine changes may simply be the functional consequence of alterations in the gene expression of genes that influence hormone signaling. In line with this argument, we observed differential regulation of several genes associated with altered gonadotropin and progesterone concentrations (Tables 3 and 4).

In this context, it should be noticed that the molecular differences between ctrl, FL1, and FL2 are only visible in sharply cycle-controlled samples. Otherwise, line-specific differences are masked by cycle-specific alterations. Second, it should be noted that the above-mentioned divergent molecular regulation mechanisms in the two high-fertility lines are not a pure black–white regulation. There are also commonly regulated genes, which are regulated in both high-fertility lines. These genes and signaling pathways, which are regulated in different ways and promote improved fertility, could offer promising targets for enhancing fertility in farm animals. Third, it should be noted that both molecular alterations (the FL1-like strategy and the FL2-like strategy) were successful in order to almost double the litter size at first delivery and therefore were successful in order to fulfill the breeding goal. However, both lines developed completely different lifetime fecundities, which was not intended and not part of the selection criteria. While FL1 remains fertile for many deliveries, FL2’s fertility is rapidly declining. Thus, we have here two high-fertility models (at first delivery) but with different lifetime fecundity. The latter aspect should be addressed in future experiments.

## Supplementary materials



## Declaration of interest

The authors declare that there is no conflict of interest that could be perceived as prejudicing the impartiality of the research reported.

## Funding

This work was supported by the German Research Foundation (DFG, MI 2098/3-1, LU 2946/1-1).

## Author contribution statement

CLML, MM, and JMW conceived the experimental design. ML was responsible for animal breeding. EKW organized the gonadotropin measurement. SB was responsible for the mRNA-Seq and conducted the statistical evaluation of the transcriptome data. CLML and MM performed the statistical evaluation of hormonal measurement and qPCR. CLML and JMW interpreted the results and drafted the manuscript. AB, US, JMW, and MM provided substantial suggestions on the interpretation and presentation of the datasets and critically read the manuscript. All authors read and approved the final manuscript.

## Data availability

Raw sequence data and raw counts were deposited at the NCBI GEO repository, entry GSE32554. Additional datasets used and/or analyzed during the current study are available from the corresponding author upon reasonable request.

## Ethics approval and consent to participate

All procedures were performed following national and international guidelines and approved by the institutional board (Animal Protection Board from the Research Institute for Farm Animal Biology). This study was conducted in accordance with ARRIVE guidelines.
