# Supplementary material for: Dampened HPG axis activity and altered ovarian gene transcription in Dummerstorf high-fertility mouse line FL2
Source: Endocr Connect. 2026 Jul 15;15(7):e260242. doi: 10.1530/EC-26-0242 (PMC13386161; doi:10.1530/EC-26-0242)
Supplement: Supplementary file 1 [file EC-26-0242_supplementary_figure.pdf]

Supplementary figure 1: Primers for reverse transcription qPCR

Gene	Primer sequence (5´-> 3´)	
	Forward	Reverse
Bmp7	CATGGTCATGAGCTTCGTCAAC	CTGGAGCACCTGATAGACTGTG
Efemp1	TAACACACCTGGTTCCTTCTACTG	TGAATGAGCCAAGAATGTTGTAGC
Rpl29	CATCCGATGACATCCGTGACTAC	TTCTTGATGCCATTTCTGTGCC
Sfrp4	AGATGCTCAAATGTGACAAGTTCC	ATTTTCATCCTCAGTGCAAACTCG
Tex14	TATCAAAAGGACCCTCCAGACTTG	AGAAGGAACATTTGCATTGTCAGG
Igfbp2	AACATCTCTACTCCCTGCACATC	CTCGTTGTAGAAGAGATGGCACT
Cxcr4	AGACTATGACTCCAACAAGGAACC	ATGCTCCTTAGCTTCTTCTGGTAA
Inhba	GAGGAGTGAACTGTTGCTATCAGA	TCCACCTCTTTCTTCTTCTTCTTG
Fshb	CAGTAGAGAAGGAAGAGTGCCG	TGTGTAGAGGGAGTCTGAGTGG
Lhb	AATGAGTTCTGCCCAGTCTGC	CACAGGCCATTGGTTGAGTCC
Gnrh	ATGATCCTCAAACTGATGGCCG	TTCTGCCATTTGATCCACCTCC
RPS18	ACCATCATGCAGAACCCACGACAGT	CAGGTCCTCACGCAGCTTGTTGTCT
36B4	AAGCGCGTCCTGGCATTGTCT	CCGCAGGGGCAGCAGTGGT
B2m	TTCTGGTGCTTGTCTCACTGAC	GCAGTTCAGTATGTTCGGCTTC
